# Using a consensus-based approach to build a threat assessment tool for use in a Canadian federal public health setting

**DOI:** 10.14745/ccdr.v52i05a01

**Published:** 2026-05-31

**Authors:** Gabrielle Brankston, Camille Dulong, Catherine Elliott, Jacqueline Middleton, Amrit Sandhu, Lindsay Whitmore

**Affiliations:** 1 Applied Public Health Sciences Directorate, Public Health Agency of Canada, Ottawa, ON

**Keywords:** public health intelligence, threat assessment method, Delphi, consensus

## Abstract

**Background:**

Public health intelligence activities, including assessment of emerging public health threats, are core operations for many public health agencies. Threat assessment is intended to guide public health actions proportional to the assessed threat level within the context of an evolving situation. A risk-based threat assessment framework helps clarify the overall population-level health impacts of threats, guiding appropriate public health action.

**Objective:**

This work aimed to develop a standardized, risk-based tool for assessing public health threats that could be used across the Public Health Agency of Canada (PHAC), integrating diverse perspectives from various PHAC stakeholders to ensure the tool is applicable across the full entire spectrum of potential public health threats.

**Methods:**

A threat assessment framework was developed to assign an overall threat level based on the assessment of three distinct threat attributes: the severity of harm to human health, the degree to which a threat is likely to impact Canada, and Canada’s capacity to prevent or mitigate the threat. A Delphi-based approach was used to develop a set of qualitative criteria enabling end-users to assign a rating of high, moderate, or low for each threat attribute and the overall assessment.

**Results:**

Three iterative surveys and two meetings led to consensus on definitions for the three threat attributes and the overall assessment, enabling a flexible framework capable of characterizing a wide range of public health threats.

**Conclusion:**

Standardized, broadly accepted definitions for threat assessment reliably characterize and communicate public health events of importance and provide support for public health action.

## Introduction

In a global environment marked by an increasing number of high-impact public health events (1), public health systems capable of rapidly detecting and assessing the significance of potential threats are essential to support decision-making by public health officials. A public health threat can be defined as verified data indicating a potential acute risk to human health that has been assessed as having the potential to harm to the health of a population (2,3). Early detection and assessment of potential public health threats is intended to guide response planning and actions proportional to the assessed level of threat within the context of an evolving situation (4). Public health actions resulting from early assessment can limit the impact of a threat on human health.

Public health intelligence activities, including the early assessment of potential public health threats, are core operations of many national and international public health agencies (5–7). Threat assessment involves characterizing a public health threat to understand its overall severity and population health impact. There is limited information in the published and grey literature about the threat assessment methodology used by public health agencies. However, public-facing reports provide an indication of the elements considered in such assessments. These include potential public health impact, severity of health effects, likelihood of exposure or transmission, and existence of control measures (1,5–7).

At the Public Health Agency of Canada (PHAC), a formalized Coordinated Threat Assessment (CTA) process was established in May 2022 in response to recommendations from the Auditor General of Canada to strengthen PHAC’s risk assessment process following an audit related to the COVID-19 pandemic (4). The CTA process sits within a spectrum of risk assessment activities, ranging from preliminary threat assessments to full, in-depth risk assessments (8). Within this spectrum, CTA coordinates technical subject-matter experts and integrates data and information from multiple sources to rapidly and systematically assess emerging public health events. This process encourages coordination of activities across PHAC and results in assessments that are standardized across programs, providing PHAC senior leadership with an overview of events that may constitute a threat to Canada and potentially require further public health action.

The original PHAC threat assessment method was designed as a user-friendly algorithm to characterize threats based on the type and urgency of federal public health actions required, such as monitoring, further investigation, or action within 48 hours. After a full year of use, along with feedback from an internal evaluation, the need was identified for a method that better captures the overall population health impact of threats to more effectively guide public health action. This led to the decision in 2023 to revise the threat assessment method to a risk-based approach.

The goal of this work was to develop a standardized tool that could be used across PHAC programs to characterize public health threats using a risk-based framework with high, moderate, and low threat levels for Canada. To address the challenge of designing a common tool that supports threat assessment across diverse populations and hazard types, a Delphi-based approach was used to gather perspectives from a range of PHAC stakeholders, ensuring applicability across the full spectrum of potential public health threats. This article describes the Delphi consensus process and presents the resulting threat assessment tool.

## Methods

**Figure 1:**
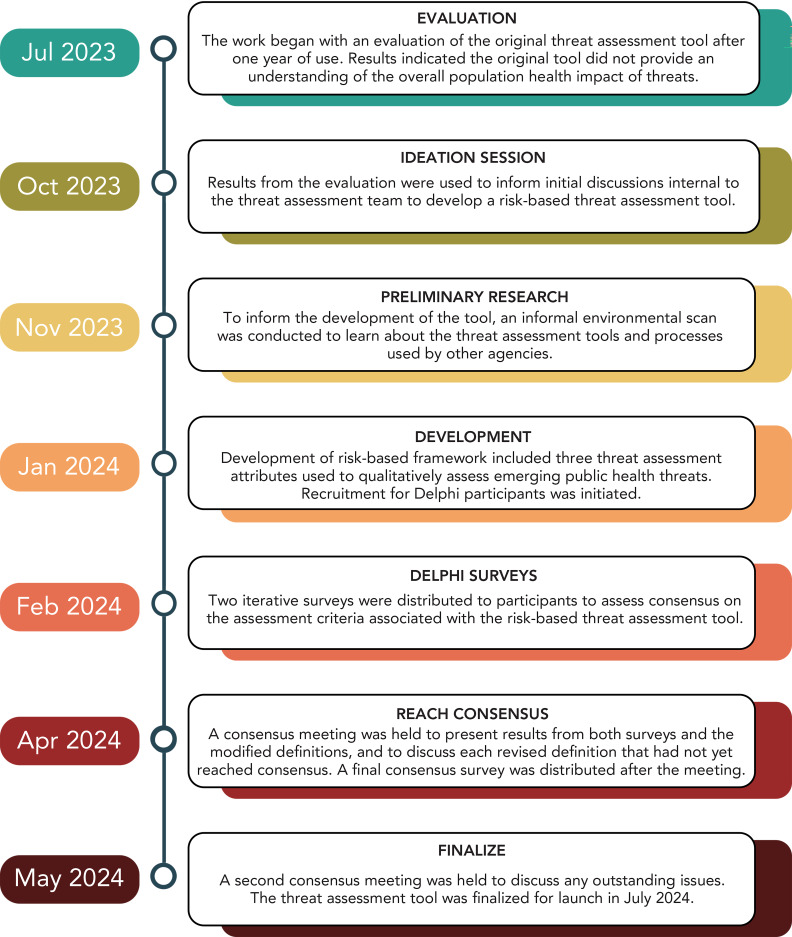
Timeline of engagement activities undertaken during the development of the threat assessment framework and methodology

Multiple steps were taken to develop and finalize the threat assessment tool. **Figure 1** depicts the activities and timeline associated with the process. Preliminary work included internal discussions within the threat assessment team, an informal environmental scan, and engagement with internal and international colleagues.

**Figure 2:**
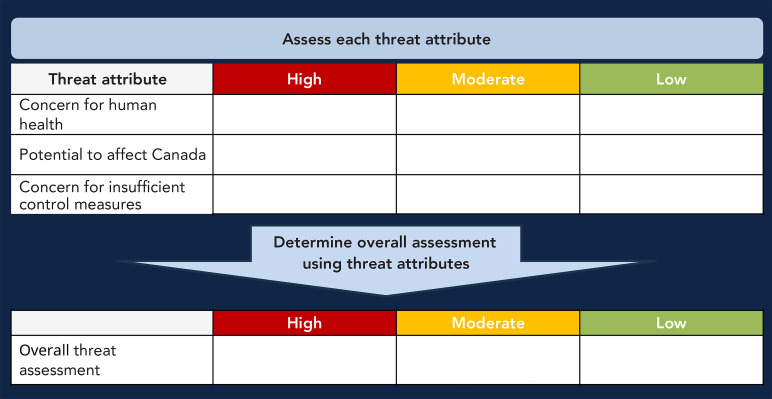
Public Health Agency of Canada’s threat assessment framework

These activities resulted in a threat assessment framework consisting of three threat attributes, each associated with three levels of assessment, and an overall assessment (**Figure 2**). The threat attributes were designed to assess the severity of harm to human health, the degree to which a threat is likely to impact Canada, and Canada’s capacity to prevent or mitigate the threat.

Once the framework was developed, a Delphi-based approach was used to define each threat attribute, the overall threat assessment, and the criteria for categorizing threats as high, moderate, and low. The Delphi technique is a scientific approach that engages experts in iterative surveys and group feedback to work towards consensus on challenging topics (9). Draft threat assessment definitions were developed by the research team to populate the framework. Consensus was defined, *a priori*, as 80% of participants in agreement with each definition, as worded.

### Participant inclusion and recruitment

Invitations to participate in the expert panel were extended to all members of PHAC’s Scientific Committee for Coordinated Threat Assessment (SCCTA). The SCCTA is a group of subject matter experts with representatives from relevant disciplines within PHAC that meet weekly to assess public health threats. Participants were actively recruited to ensure representation from all stakeholder groups, reflecting the end-users of the framework for threat assessment from a national perspective.

### Surveys

Agreement with definitions was assessed through three rounds of surveys conducted online using Microsoft Forms. Survey data were collected anonymously, and no directly identifying information was collected from participants. For the first two surveys, agreement with each definition was assessed using a five-point Likert scale with the following options: Yes, as currently worded; Yes, but change wording; Unsure; Probably not; and Definitely not. Respondents were also asked to provide detailed feedback or suggest alternative wording, which was used to inform modifications to the definitions.

Participants were given eight business days to complete each survey round. Two reminders were sent to participants for each survey. After each survey round, all participant feedback was considered in revising the definitions. Participants were then provided a summary of the results, reasons for changes to the definitions, and the next iteration of the definitions. Subsequent surveys and the consensus meeting included only items that had not reached 80% agreement. Following the initial consensus meeting, a third survey was conducted to formally assess agreement using a binary response format (i.e., yes/no).

### Consensus meetings

Two consensus meetings were held by videoconference three weeks apart and were facilitated by a member of the research team. Results from both surveys and the modified definitions were presented at the first consensus meeting. Participants discussed each revised definition that had not reached consensus after the second survey. Areas of disagreement were explored, and definitions were further modified as needed. Consensus on each item discussed during the meeting was assessed using a post-meeting survey, with consensus defined as 80% agreement. A second consensus meeting was conducted to address outstanding areas of disagreement and to discuss the process by which the tool would be operationalized.

### Data analysis

For each iteration of the survey, quantitative data were analyzed using simple descriptive statistics (i.e., the proportion of participants selecting each level of agreement) using R Studio (10). A qualitative thematic analysis (11) was used to summarize free text data into broad categories, informing feedback to participants and revisions to the definitions.

## Results

### Participants

A total of 26 participants were included in the consensus process. Of the 10 PHAC program areas that regularly contribute to threat assessment, representatives from eight agreed to participate, along with additional members of the SCCTA, resulting in representation from 12 programs in total. Representatives included subject matter experts in zoonotic disease (n=1), outbreak management and food safety (n=2), respiratory virus epidemiology and surveillance (including COVID-19) (n=4), risk assessment (n=8), travel health (n=3), vaccine surveillance and readiness (n=2), emergency management (n=1), biosecurity (n=1), public health measures (n=2), risk sciences and modelling (n=1), and International Health Regulations (n=1).

### Surveys

Of the 26 participants, there were 23 responses to survey 1 (88.5%), 19 responses to survey 2 (73.1%), and 10 responses to survey 3 (38.5%). Over the course of the study, there was a progressive, positive shift toward consensus with iterative modifications of the definitions (**Table 1**).

**Table 1:** Proportion of respondents in agreement with each definition for each of three Delphi surveys

Threat assessment item	Proportion of respondents in agreement with each definition as worded		
**Definition**	**Survey 1** **(n=23)**	**Survey 2** **(n=19)**	**Survey 3** **(n=10)**
**Concern for human health**	**43.5%**	**78.9%**	**100%**
High	52.2%	78.9%	100%
Moderate	60.9%	78.9%	100%
Low	60.9%	78.9%	100%
**Potential to affect Canada**	**34.8%**	**68.4%**	**90.0%**
High	39.1%	78.9%	90.0%
Moderate	34.8%	73.7%	90.0%
Low	39.1%	78.9%	90.0%
**Concern for insufficient control measures**	**52.2%**	**84.2%**	**N/A^a^**
High	69.6%	78.9%	90.0%
Moderate	47.8%	68.4%	90.0%
Low	73.9%	73.7%	90.0%
**Overall threat assessment**	**69.6%**	**84.2%**	**N/A^a^**
High	69.6%	78.9%	Not assessed
Moderate	69.6%	78.9%	Not assessed
Low	69.6%	78.9%	Not assessed

Abbreviation: N/A, not applicable

^a^ Reached 80% consensus on survey 2

No definitions achieved consensus in the first survey, two achieved consensus in the second, and all definitions assessed after the consensus meetings reached 90% to 100% agreement, exceeding the 80% target. The definitions for high, moderate, and low overall threat assessment were not assessed in the final survey however, verbal agreement amongst attendees was achieved during the second consensus meeting. Thematic analysis of the written feedback resulted in categorization by the following broad themes: wording changes, framework language, and clarifications.

### Consensus meetings

The first and second consensus meetings were attended by 15 (57.7%) and 17 (65.4%) participants, respectively, representing 11 of the 12 participating programs. Participants discussed and refined the definitions, raised methodological questions and program-specific issues about how to apply the resulting definitions, and commented on practical considerations for applying the tool (e.g., assessing international threats not yet present in Canada). These discussions prompted group exploration of related operational challenges. Participants informally reported a deeper understanding and greater level of comfort with the tool after engaging in these conversations. The final threat assessment definitions are presented in **Table 2**.

**Table 2:** Final definitions for the threat assessment tool

Item	Definition
**Concern for human health**	**The potential severity of harm to health that the hazard poses in the population most likely to be affected, based on current knowledge.**
High	This hazard poses or is anticipated to pose severe harm to the health of the population most likely to be affected (e.g., a hazard that is typically life-threatening and/or involves permanent or long-term harmful health consequences).
Moderate	This hazard poses or is anticipated to pose moderate harm to the health of the population most likely to be affected (e.g., a hazard that is typically associated with serious but not life-threatening health effects or typically involves non-permanent or short-term harmful health consequences).
Low	This hazard poses or is anticipated to pose minor harm to the health of the population most likely to be affected (e.g., a hazard that is typically self-limiting or associated with minimal harmful health consequences).
**Potential to affect Canada**	**The degree to which the hazard is likely to affect the health of people in Canada, entering Canada, and/or Canadian residents abroad, based on knowledge at the time of assessment.**
High	The hazard is currently affecting or is likely to affect large numbers^a^ of people in the general population or in specific subgroups in Canada, entering Canada, and/or Canadian residents abroad.
Moderate	The hazard is currently affecting or is likely to affect a moderate number^a^ of people in the general population or in specific subgroups in Canada, entering Canada, and/or Canadian residents abroad.
Low	The hazard is currently affecting or is likely to affect a minimal number^a^ of people in the general population or in specific subgroups in Canada, entering Canada, and/or Canadian residents abroad. OR The hazard is unlikely to affect people in Canada, entering Canada, and/or Canadian residents abroad.
**Concern for insufficient control measures**	**The potential that Canada has insufficient measures to detect, prevent, mitigate, prepare for, and/or respond to the hazard or associated negative health impact(s).**
High	There are currently no known mechanisms to detect, prevent, mitigate, prepare for, and/or respond to the impact(s) of this hazard on Canada^b^. OR There may be limited mechanisms to detect, prevent, mitigate, prepare for, and/or respond to the impact(s) of this hazard on Canada^b^, but they are considered experimental, of unknown effectiveness, or unavailable. OR Necessary measures will require significant resources to implement.
Moderate	There are mechanisms to detect, prevent, mitigate, prepare for, and/or respond to the impact(s) of this hazard on Canada^b^, but there may be challenges with effectiveness, availability, or deployment. OR Necessary measures may require increased resources to implement.
Low	There are effective, available, and easily deployed mechanisms to detect, prevent, mitigate, prepare for, and/or respond to the impact(s) of this hazard on Canada^b^. OR Routine responses are adequate (i.e., there is no need to implement additional control measures).
**Overall threat assessment**	**The overall evaluation of the threat posed by the hazard associated with this signal, at the time of assessment, taking into consideration ‘Concern for human health’, ‘Potential to affect Canada’, and ‘Concern for insufficient control measures’.**
High	The hazard associated with this signal is assessed at a high threat level for Canada, at the time of assessment.
Moderate	The hazard associated with this signal is assessed at a moderate threat level for Canada, at the time of assessment.
Low	The hazard associated with this signal is assessed at a low threat level for Canada, at the time of assessment.

^a^ There are no quantitative guidelines for this attribute. The number of people potentially or currently affected that would be considered high, moderate, or low will vary depending on the context of the event

^b^ Canada refers to people living in Canada, entering Canada, and/or Canadian residents abroad

## Discussion

The new threat assessment framework was designed to assess the severity of harm to human health, the degree to which a threat is likely to impact Canada, and Canada’s capacity to prevent or mitigate the threat. This article describes the Delphi consensus process used to determine a set of qualitative criteria with which users will assign a rating of high, moderate, or low for each of the three distinct threat attributes. Engaging a range of subject-matter experts led to consensus on 16 definitions in three distinct categories and one overall assessment category, resulting in a flexible framework capable of characterizing a wide range of public health threats.

The framework is aligned with other national and international health agencies in terms of the important elements to consider in public health threat assessment (5–7) and qualitative flexibility (5). Moving from the previous algorithm-based approach to a qualitative framework allows for flexibility in weighting individual threat attributes according to their relative importance in the context of a specific threat. Unlike the former algorithm-based approach, which based assessments on federal actions, the new risk-based characterization provides a clearer representation of the key information used to assess a threat and can better inform appropriate public health actions. A separate publication provides an in-depth description of the threat assessment process at PHAC and includes an example of threat assessment in practice (12).

Several strengths were associated with using a Delphi-based approach to develop definitions for a threat assessment tool. Achieving consensus among experts from multiple subject-matter areas provides confidence that the process produced a robust tool capable of appropriately characterizing public health threats and that is applicable to multiple areas of public health. Including consensus meetings allowed participants to provide verbal feedback, which resulted in greater clarification of their views than was possible with surveys alone. This collaborative process fostered understanding and acceptance of the definitions across programs. Finally, programs expressed appreciation for the opportunity to participate in this process and contribute to the development of the tool.

### Limitations

The process of developing the new threat assessment tool had several limitations. The initial steps to develop the framework were conducted within the research team and, while based on feedback from programs and an international scan, may have been designed differently if a process of systematic feedback had been used. Participation declined by 57% over the course of the Delphi study, and the anonymous nature of the surveys made it impossible to assess representativeness across PHAC programs. However, participation in the consensus meetings was largely representative of the complete list of participating programs, including programs that contribute regularly to the threat report. Finally, in terms of the Delphi methodology, the statistical stability of responses was not measurable because only two rounds of surveys were conducted (13).

## Conclusion

Standardized, broadly accepted definitions for threat assessment in a federal public health setting are key to reliably characterizing and communicating public health events of importance and providing support for public health action. Ongoing work in this area will lead to more reliable and valid assessments of a variety of threats in multiple public health settings. Future plans include validating and evaluating the threat assessment tool, as well as continuing to apply it to threats beyond infectious diseases.
